# Supplementary material for: Autoimmune non-coding variants perturb transcription factor–cofactor complex assembly linked to enhancer activity
Source: bioRxiv. 2026 May 22:2026.05.20.726379. Preprint. [Version 1] doi: 10.64898/2026.05.20.726379 (PMC13228337; doi:10.64898/2026.05.20.726379)
Supplement: Supplement 6 [file NIHPP2026.05.20.726379v1-supplement-6.pdf]

bioRxiv preprint doi: https://doi.org/10.64898/2026.05.20.726379; this version posted May 22, 2026. The copyright holder for this preprint (which was not certified by peer review) is the author/funder, who has granted bioRxiv a license to display the preprint in perpetuity. It is made available under a CC-BY 4.0 International license.

# Supplementary Figures

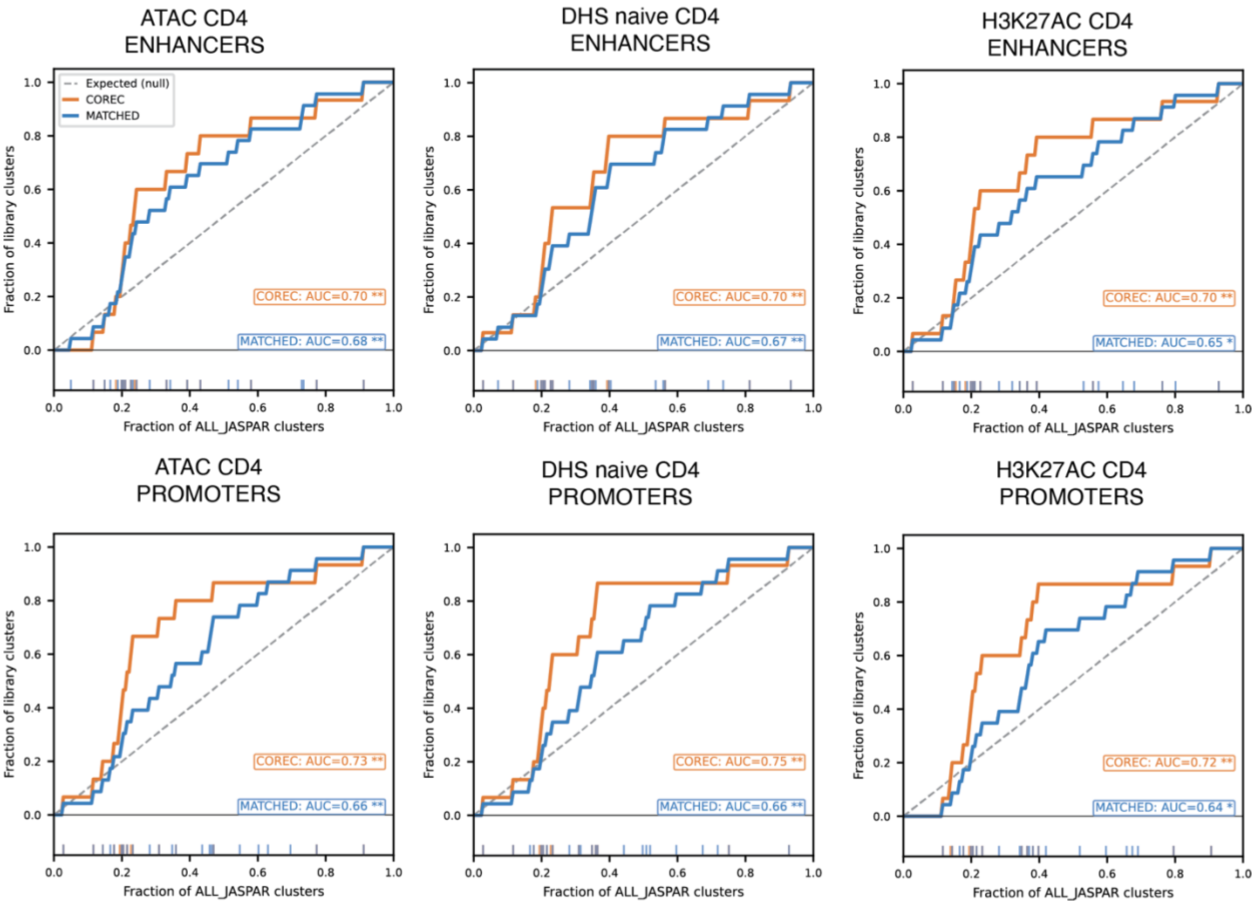


**Supplementary Figure 1. Top CASCADE motif clusters are enriched in CD4+ T-cell regulatory elements.**
ROC-style enrichment curves showing the rank enrichment of CoRec and matched motif clusters across CD4+ T-cell regulatory element datasets defined by ATAC-seq, naive CD4 DHS, and H3K27ac marks. Motif clusters were ranked using HOMER enrichment scores across all JASPAR clusters. The x-axis shows the cumulative fraction of all JASPAR clusters ranked by score, and the y-axis shows the cumulative fraction of library clusters seen. Separate panels show enhancer and promoter annotations. The dashed line indicates the expected null distribution. AUC values are shown for CoRec and matched motif-cluster rankings.

bioRxiv preprint doi: https://doi.org/10.64898/2026.05.20.726379; this version posted May 22, 2026. The copyright holder for this preprint (which was not certified by peer review) is the author/funder, who has granted bioRxiv a license to display the preprint in perpetuity. It is made available under a CC-BY 4.0 International license.

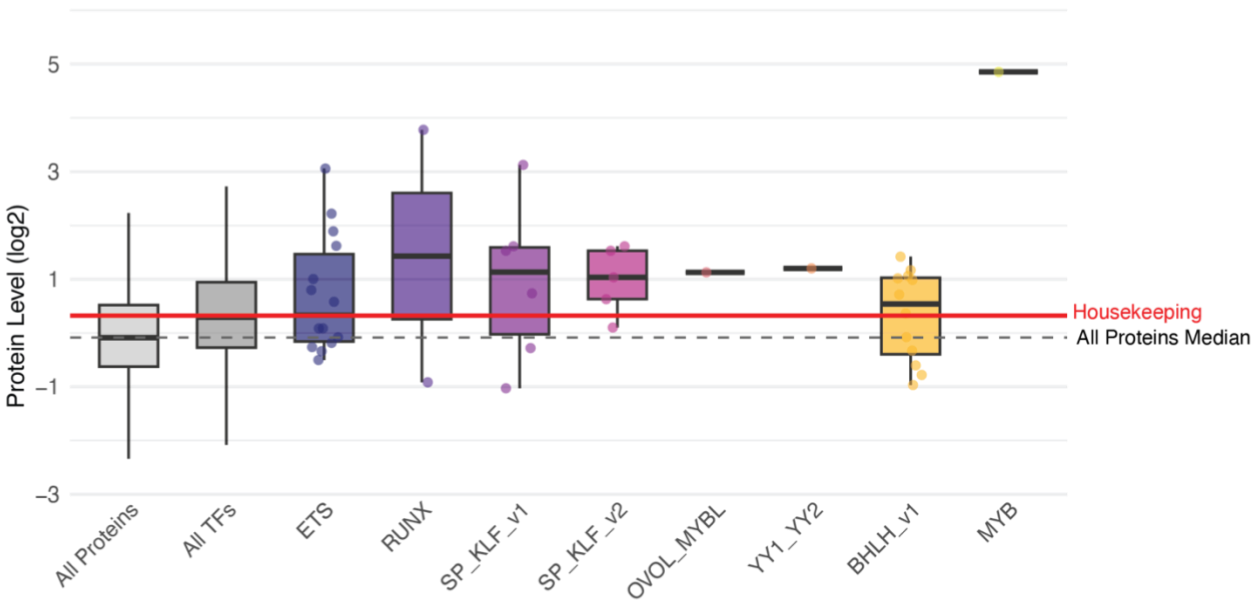


**Supplementary Figure 2. Protein abundance of TFs from top CASCADE motif clusters in Jurkat cells.**

Boxplots showing normalized protein abundance distributions for TFs assigned to major CASCADE-identified motif clusters in Jurkat cells, compared with all detected proteins and all detected TFs. Each overlaid point represents an individual TF member within the indicated motif cluster. Protein levels are shown on a log2 scale, and the housekeeping protein median is shown as a reference.

bioRxiv preprint doi: https://doi.org/10.64898/2026.05.20.726379; this version posted May 22, 2026. The copyright holder for this preprint (which was not certified by peer review) is the author/funder, who has granted bioRxiv a license to display the preprint in perpetuity. It is made available under a CC-BY 4.0 International license.

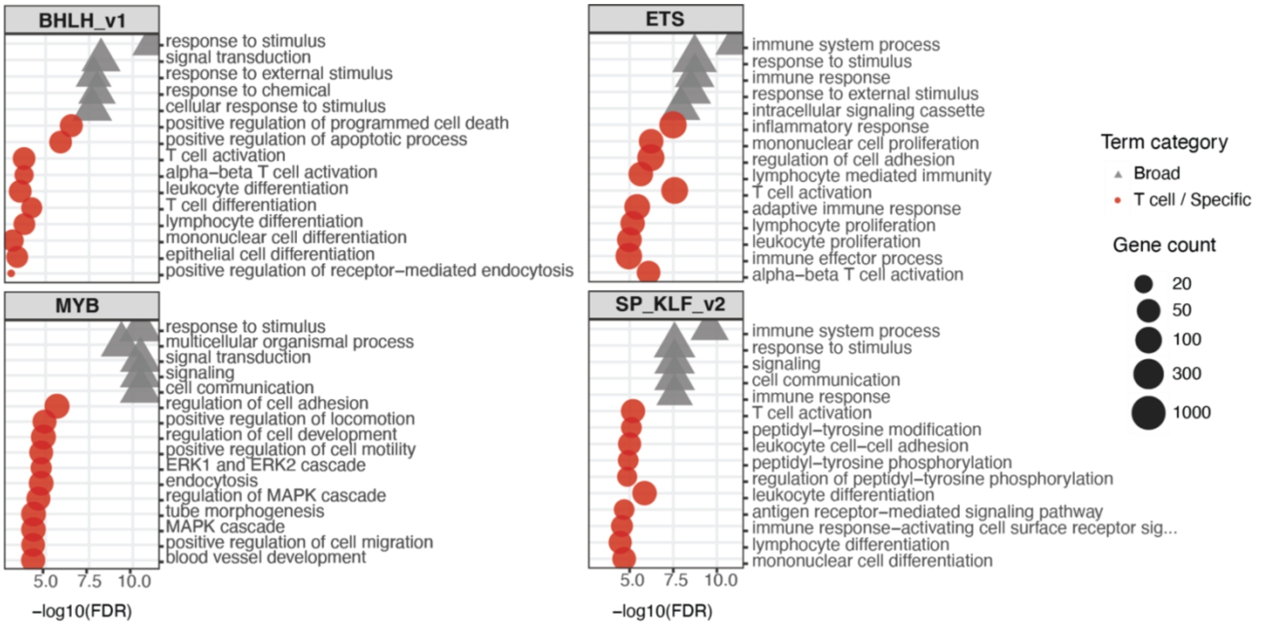


**Supplementary Figure 3. Perturb-seq GO enrichment supports T-cell regulatory roles for dominant CASCADE motif families.**

GO Biological Process enrichment analysis of genes differentially expressed after knockdown of TF-family members in resting primary CD4+ T cells. Enrichment results are shown for BHLH_v1, ETS, MYB, and SP_KLF_v2 families. Terms are categorized as broad biological processes or T-cell/specific immune-related processes. Point size indicates the number of genes associated with each term, and the x-axis shows −log10(FDR).
